# Supplementary material for: The evolution of CHROMOMETHYLASES and gene body DNA methylation in plants
Source: Genome Biol. 2017 May 1;18:65. doi: 10.1186/s13059-017-1195-1 (PMC5410703; doi:10.1186/s13059-017-1195-1)
Supplement: Supplementary file 2 — Figures S1 to S11 with legends. (PDF 23575 kb) [file 13059_2017_1195_MOESM2_ESM.pdf]

Figure S1. Syntenic relationships support a Whole Genome Duplication (WGD) event giving rise to CMT2 and CMT3/ZMET. Collinearity was determined using CoGe's GEvo program, and is indicated by connected blocks. Collinearity is more pronounced in some species over others, which suggests sequence divergence following the shared WGD placed at the base of all angiosperms [36]. Yellow blocks indicate the location of CMT2 or CMT3/ZMET.

Figure S2. CMT proteins in green algae (*C. reinhardtii*, *Chlorella sp.* NC64A, and *V. carteri*) might represent misidentified homologs. a, A midpoint rooted gene tree constructed from a subset of species and green algae using protein sequences. Previously identified CMT homologs in *C. reinhardtii*, *Chlorella sp.* NC64A, and *V. carteri* (JGI accession ids 190580, 52630, and 94056, respectively) have low amino acid sequence similarity to *A. thaliana* CMT compared to other green algae species (Additional file ###: Table S1), which is reflected in long branches, especially for *C. reinhardtii* and *V. carteri*. Values on branches are raw branch lengths represented as amino acid substitutions per amino acid site. **b**, Protein structure of previously identified CMT homologs in *C. reinhardtii*, *Chlorella sp.* NC64A, and *V. carteri* and those identified in green algae from the 1KP dataset. Reported CMTs in *C. reinhardtii* and *Chlorella sp.* NC64A do not contain CHROMO domains, and the homolog in *V. carteri* does not contain any recognizable PFAM domains, however BAH, CHROMO and a DNA methylase domain can all be identified in green algae CMT homologs from the 1KP dataset.

Figure S3. Phylogenetic relationships among CMTs in Viridiplantae. CMTs are separated into four monophyletic clades based on bootstrap support and the relationship of *A. thaliana* CMTs: (i) the gbM-dependent CMT superclade with subclades CMT1, CMT3, ZMET and *A. trichopoda*; (ii) CMT2 and; (iii) homologous (hCMT) α and β. Values at nodes in represent bootstrap support from 1000 replicates, and the tree was rooted to the clade containing all green algae species.

Figure S4. Syntenic relationships support a WGD event giving rise to CMT1 and CMT3 in eudicots and ZMET paralogs. a, Collinearity was determined using CoGe's GEvo program, and is indicated by connected blocks. Collinearity is more pronounced in some eudicots over others, which suggests sequence divergence following the shared WGD placed at the base of all eudicots [36]. b, Phylogenetic relationships of ZMETs in the Poaceae suggest WGD events are shared by several species and are species-specific as is the case for ZMET2 and ZMET5 in Z. mays. Colors following the tip labels indicate clades of paralogous ZMETs. c, Similarly to eudicots, WGD is supported by collinearity upstream and downstream of ZMET paralogs. Yellow blocks indicate the location of CMT1 or CMT3/ZMET.

Figure S5. Presence and absence of CMTs and ZMETs in eudicots, and monocots and monocots/commelinids, respectively. **a**, Eudicot (basal, core, rosid, and asterid) species of plants possess different combinations of CMT1, CMT2, and CMT3. CMT3 was potentially loss from 46/262 (18%), and CMT1 is found in 106/262 (40%) of eudicot species sequenced by the 1KP Consortium. Species without CMT3 are predicted to have significantly reduced levels of gbM loci compared to eudicot species with CMT3. The presence of CMT1 in numerous species suggests a yet to be determined functional role of CMT1 in DNA methylation and/or chromatin modification. **b**, Similarly to eudicots, monocots and monocots/commelinids have different combinations of CMT2 and ZMET, which may reflect differences in genome structure, and DNA methylation and chromatin modification patterns.

Figure S6. Metagene plots of DNA methylation across gene bodies. DNA methylation levels within all full-length coding sequences or transcripts for additional species used in this study.

Figure S7. MCG-enriched genes in species sister to angiosperms are rare and not strongly conserved. **a**, The proportion of mCG-enriched genes are variable across Embryophyta. However, lowest levels are seen in species null for CMT3 and that possess a non-orthologous CMT3 (white circles). Additionally, species that possess a CMT3 that has experienced elevated rates of evolution (ω) have a lower proportion of mCG-enriched genes (gray circles). **b**, The majority of mCG-enriched genes are orthologous to non-mCG-enriched genes in A. thaliana or have no hits to an *A. thaliana* gene based on an e-value of ≤1E-06. However, *P. taeda* is an exception, which suggests some of the mCG-enriched genes are conserved to gbM genes in A. thaliana.

Figure S8. MCG in genes of species sister to angiosperms are biased towards extreme low or high levels. Distributions of mCG across all genes with sufficient coverage for species with sequenced genomes (see Methods).

Figure S9. Jumonji (jmjC) domain-containing gene family phylogeny. The jmjC domain-containing family contains five monophyletic clades based on the location of *A. thaliana* genes. Only angiosperm sequences can be found within the clade containing *A. thaliana* IBM1. Scale bar represents nucleotide substitutions per site.

Figure S10. SUVH4 and SUVH5/6 gene family phylogenies. a, SUVH4 gene family approximately recapitulate species relationships, and angiosperm-specific monophyletic clades are not observed based on bootstrap support and the placement of A. thaliana SUVH4. **b**, However, Brassicaceae-specific monophyletic clades delineate SUVH5 and SUVH6, hence a homologous SUVH5/6 (hSUVH5/6) sequence is found in other Embryophyta. However, some nodes – especially those delineating monocot and monocot/commelinid hSUVH5/6 sequences – are weakly supported. **c**, Phylogenetic relationships support a Brassicacae-specific duplication event, which gave rise to SUVH5 and SUVH6. **d**, Reanalyzing monocot and monocot/commelinid hSUVH5/6 sequences increases bootstrap support delineating two monophyletic clades. This relationship is analogous to SUVH5 and SUVH6 in Brassicaceae, but encompasses all monocots and monocot/commelinids. Furthermore, Poaceae-specific monophyletic clades are observed within each of the monocot- and monocot/commelinid-specific monophyletic clades. Phylogenetic relationships support multiple duplication events in the monocots and monocot/commelinids. Values at nodes represent bootstrap support and scale bar represents nucleotide substitutions per site.

Figure S11. A linear model to determine DNA methylation levels from low sequence coverage WGBS. A strong linear correlation is observed between DNA methylation levels at CG, CHG and CHH sites determined from low, subsampled and full WGBS coverage. A linear model was generated for each sequence context, which was used to extrapolate levels of DNA methylation from species with low WGBS coverage. Each data point represents a single plant species from [19, 20, 25, 40].

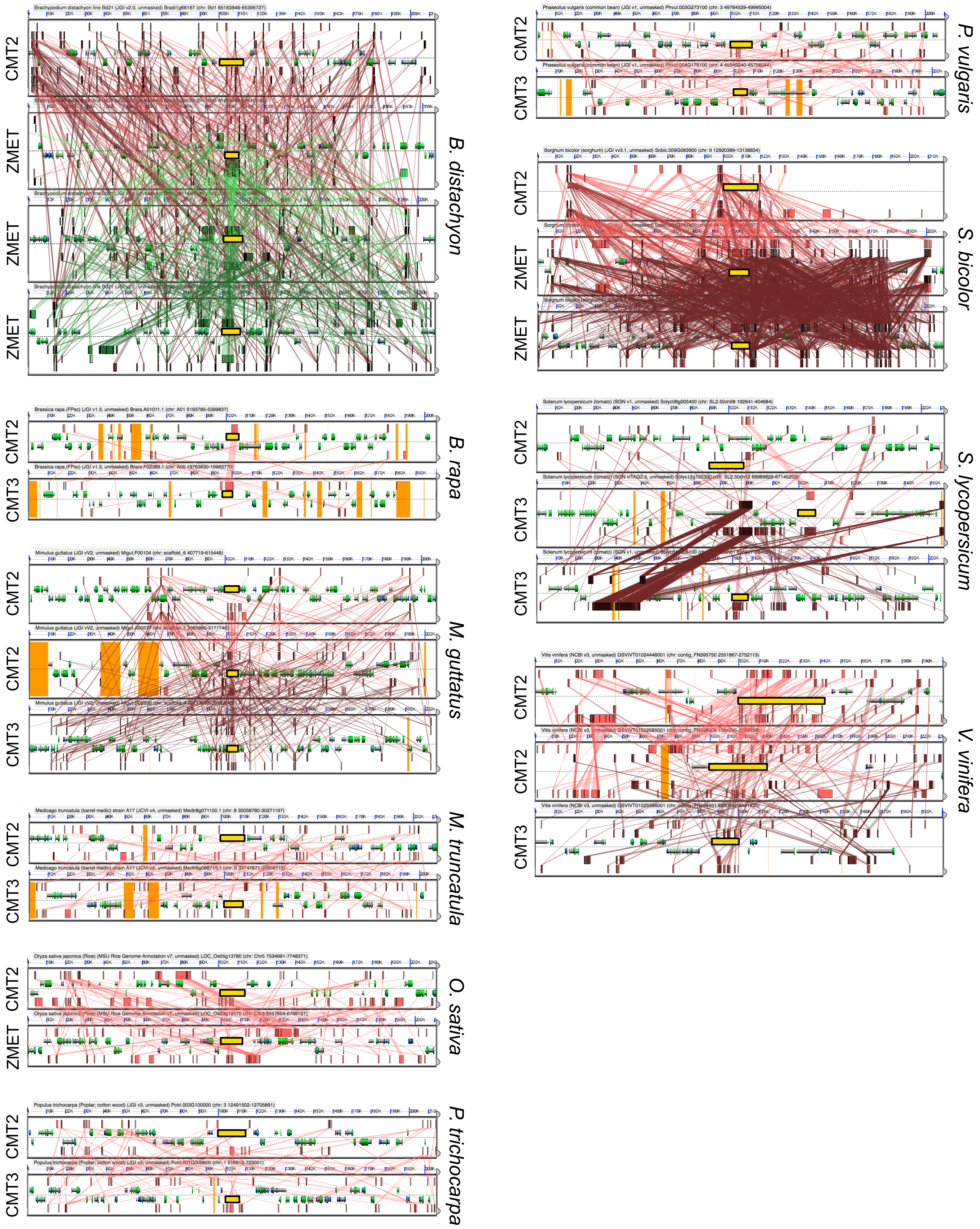

B. distachyon
CMT2
ZMET
ZMET
ZMET
B. rapa
CMT2
CMT3
M. guttatus
CMT2
CMT2
CMT3
M. truncatula
CMT2
CMT3
O. sativa
CMT2
ZMET
P. trichocarpa
CMT2
CMT3
P. vulgaris
CMT2
CMT3
S. bicolor
CMT2
ZMET
ZMET
S. lycopersicum
CMT2
CMT3
CMT3
V. vinifera
CMT2
CMT2
CMT3

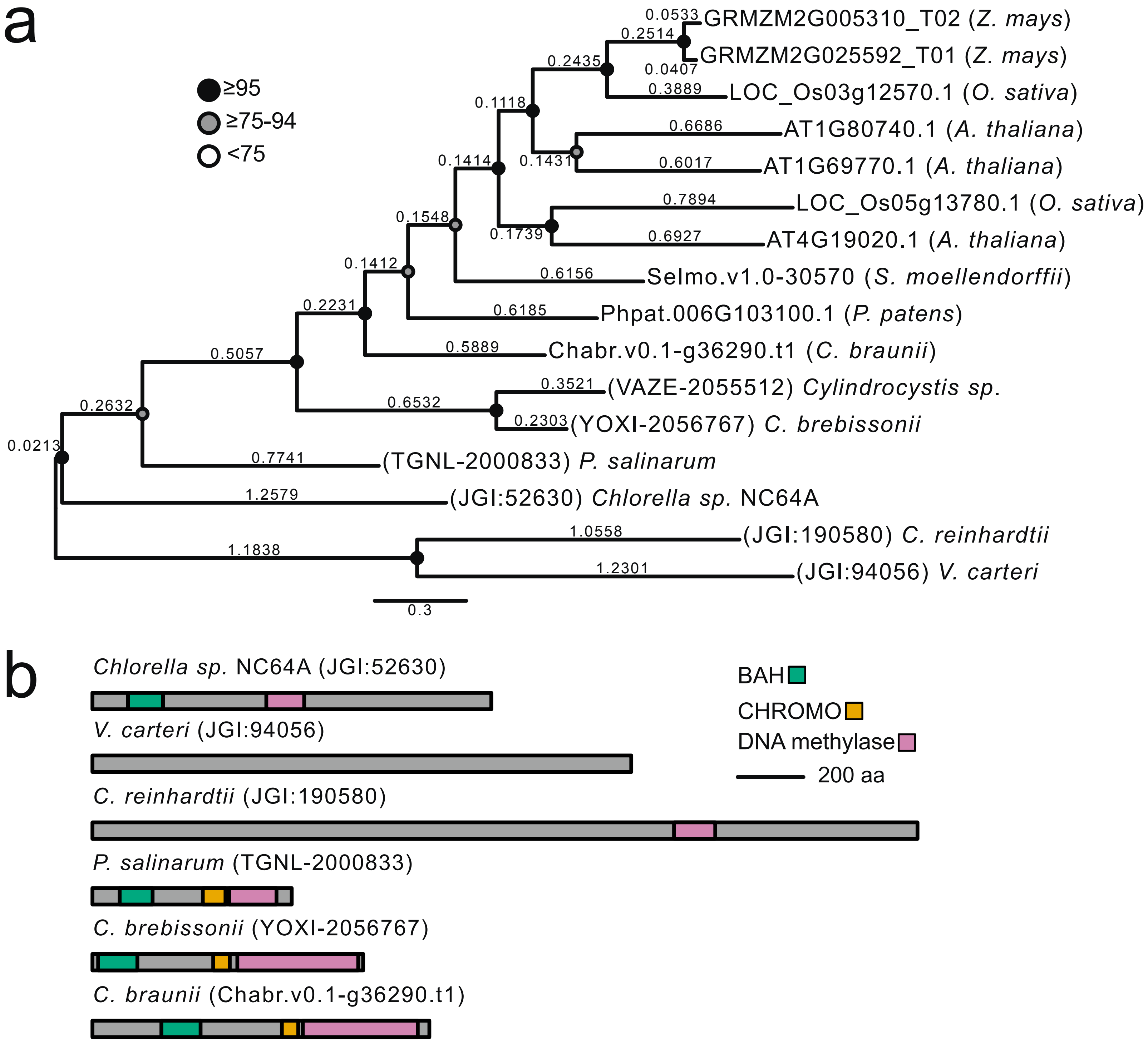

a
≥95
≥75-94
<75
GRMZM2G005310_T02 (Z. mays)
GRMZM2G025592_T01 (Z. mays)
LOC_Os03g12570.1 (O. sativa)
AT1G80740.1 (A. thaliana)
AT1G69770.1 (A. thaliana)
LOC_Os05g13780.1 (O. sativa)
AT4G19020.1 (A. thaliana)
Selmo.v1.0-30570 (S. moellendorffii)
Phpat.006G103100.1 (P. patens)
Chabr.v0.1-g36290.t1 (C. braunii)
(VAZE-2055512) Cylindrocystis sp.
(YOXI-2056767) C. brebissonii
(TGNL-2000833) P. salinarum
(JGI:52630) Chlorella sp. NC64A
(JGI:190580) C. reinhardtii
(JGI:94056) V. carteri
0.0533
0.2514
0.0407
0.2435
0.3889
0.1118
0.6686
0.1431
0.6017
0.1414
0.7894
0.1739
0.6927
0.1548
0.6156
0.1412
0.6185
0.2231
0.5889
0.5057
0.3521
0.6532
0.2303
0.2632
0.7741
0.0213
1.2579
1.0558
1.1838
1.2301
0.3
b
Chlorella sp. NC64A (JGI:52630)
V. carteri (JGI:94056)
C. reinhardtii (JGI:190580)
P. salinarum (TGNL-2000833)
C. brebissonii (YOXI-2056767)
C. braunii (Chabr.v0.1-g36290.t1)
BAH
CHROMO
DNA methylase
200 aa

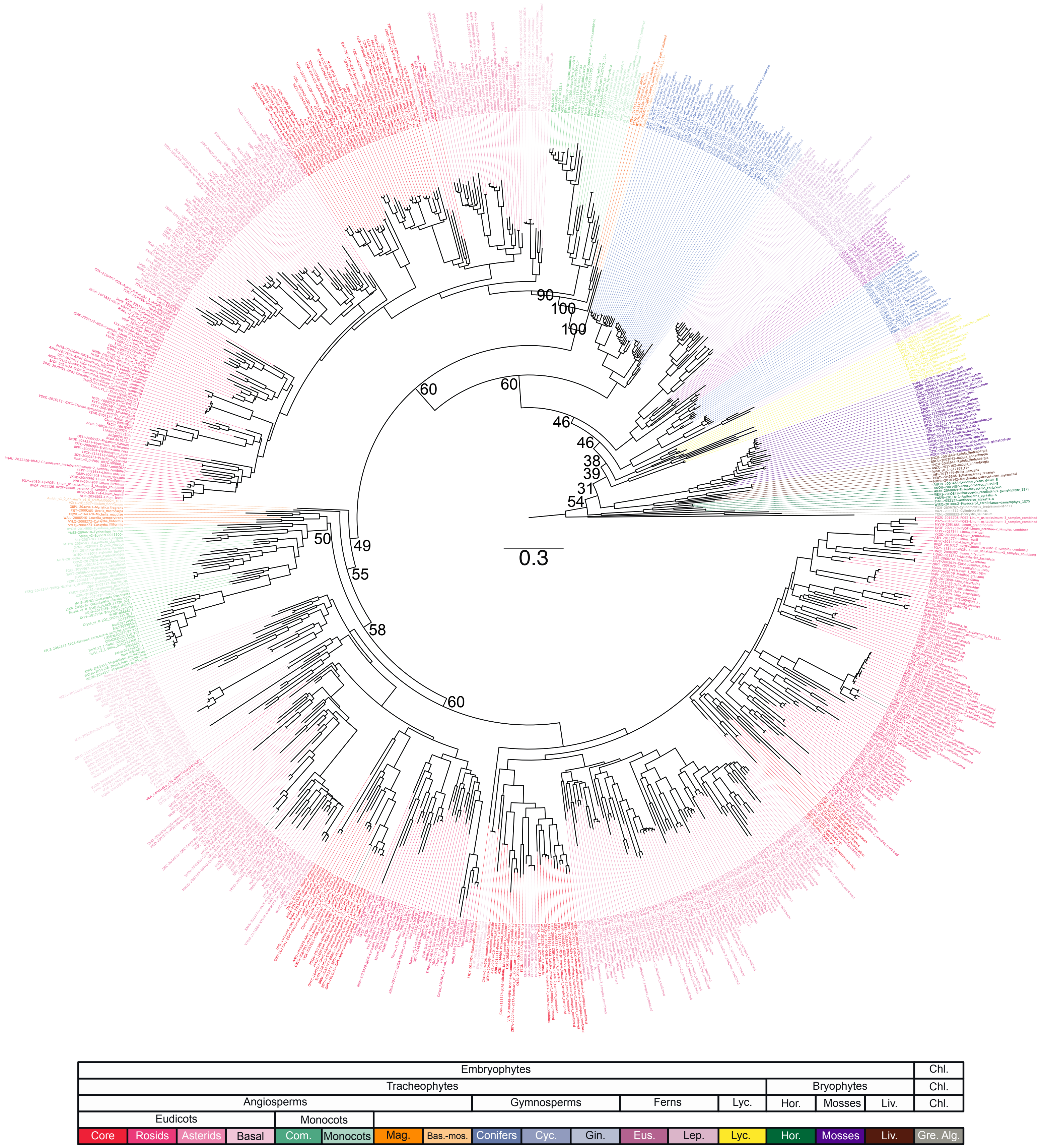

90
100
100
60
60
46
46
38
39
31
54
50
49
55
58
60
0.3
Embryophytes
Chl.
Tracheophytes
Bryophytes
Chl.
Angiosperms
Gymnosperms
Ferns
Lyc.
Hor.
Mosses
Liv.
Chl.
Eudicots
Monocots
Core
Rosids
Asterids
Basal
Com.
Monocots
Mag.
Bas.-mos.
Conifers
Cyc.
Gin.
Eus.
Lep.
Lyc.
Hor.
Mosses
Liv.
Gre. Alg.

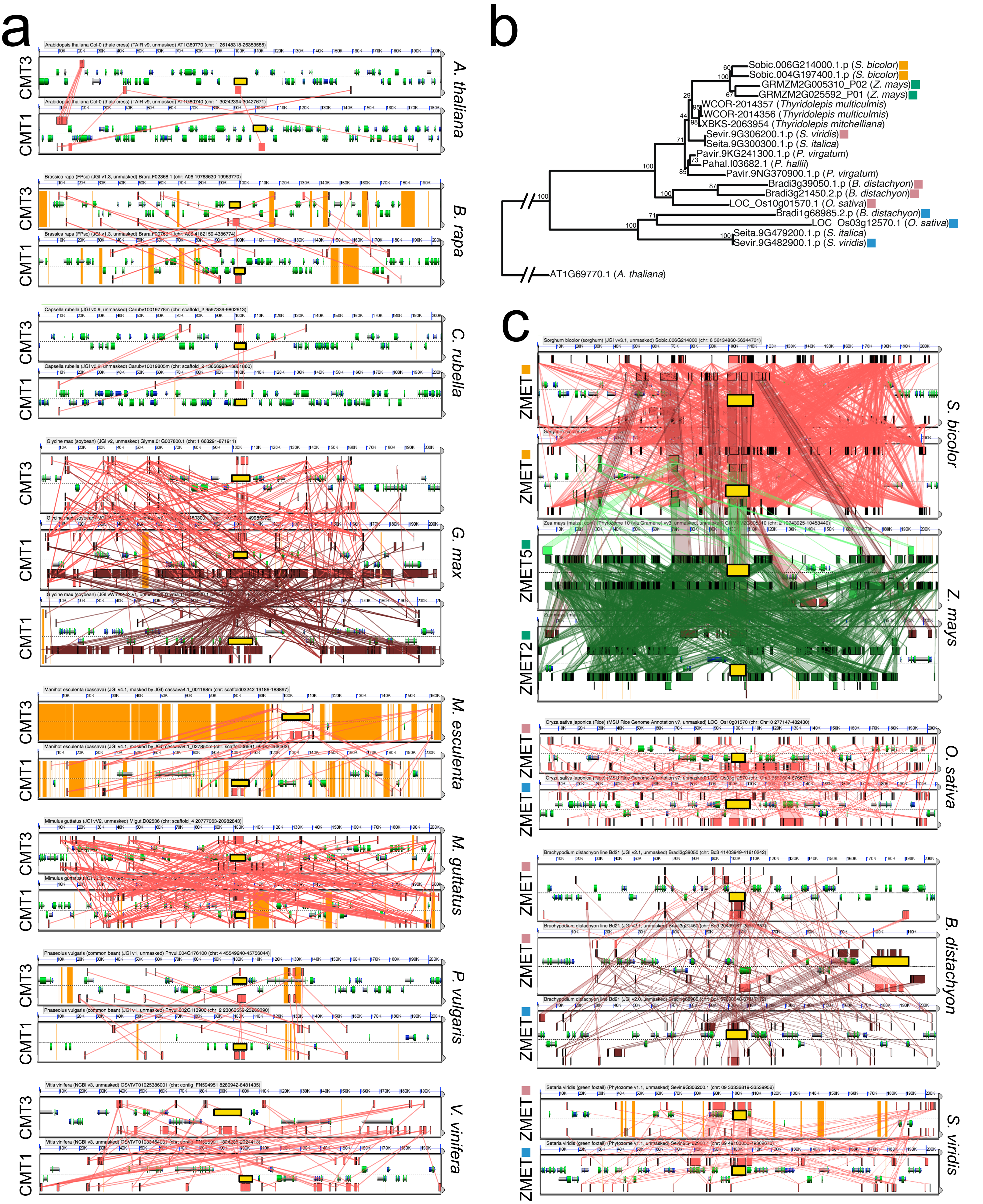

a
CMT3
CMT1
A. thaliana
B. rapa
C. rubella
G. max
M. esculenta
M. guttatus
P. vulgaris
V. vinifera
b
Sobic.006G214000.1.p (S. bicolor)
Sobic.004G197400.1.p (S. bicolor)
GRMZM2G005310_P02 (Z. mays)
GRMZM2G025592_P01 (Z. mays)
WCOR-2014357 (Thyridolepis multiculmis)
WCOR-2014356 (Thyridolepis multiculmis)
XBKS-2063954 (Thyridolepis mitchelliana)
Sevir.9G306200.1.p (S. viridis)
Seita.9G300300.1.p (S. italica)
Pavir.9KG241300.1.p (P. virgatum)
Pahal.I03682.1 (P. hallii)
Pavir.9NG370900.1.p (P. virgatum)
Bradi3g39050.1.p (B. distachyon)
Bradi3g21450.2.p (B. distachyon)
LOC_Os10g01570.1 (O. sativa)
Bradi1g68985.2.p (B. distachyon)
LOC_Os03g12570.1 (O. sativa)
Seita.9G479200.1.p (S. italica)
Sevir.9G482900.1.p (S. viridis)
AT1G69770.1 (A. thaliana)
60
100
67
29
95
44
98
71
73
85
100
87
100
100
71
100
100
c
ZMET
ZMET5
ZMET2
S. bicolor
Z. mays
O. sativa
B. distachyon
S. viridis

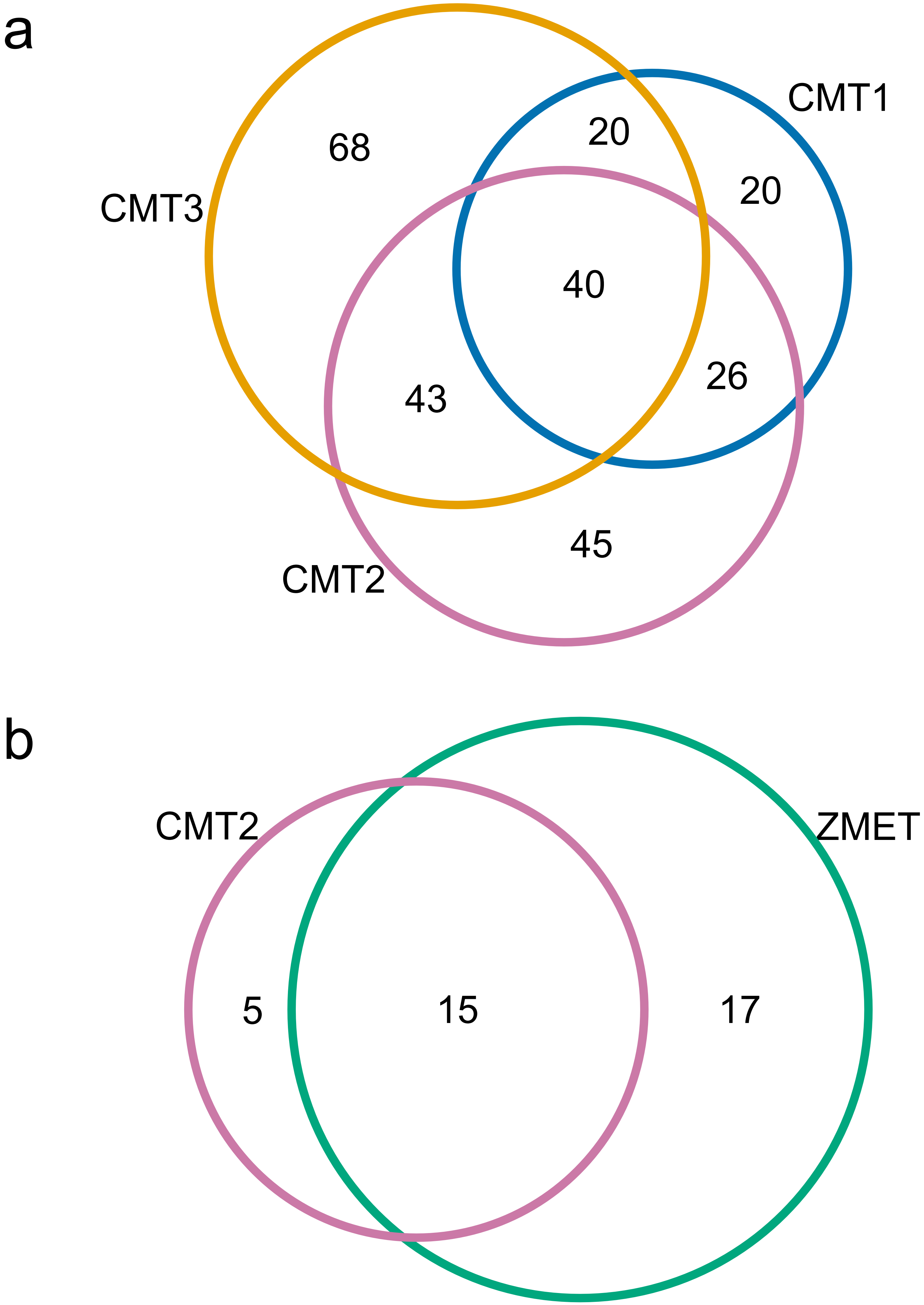
a
CMT1
CMT3
CMT2
68
20
20
40
43
26
45
b
CMT2
ZMET
5
15
17

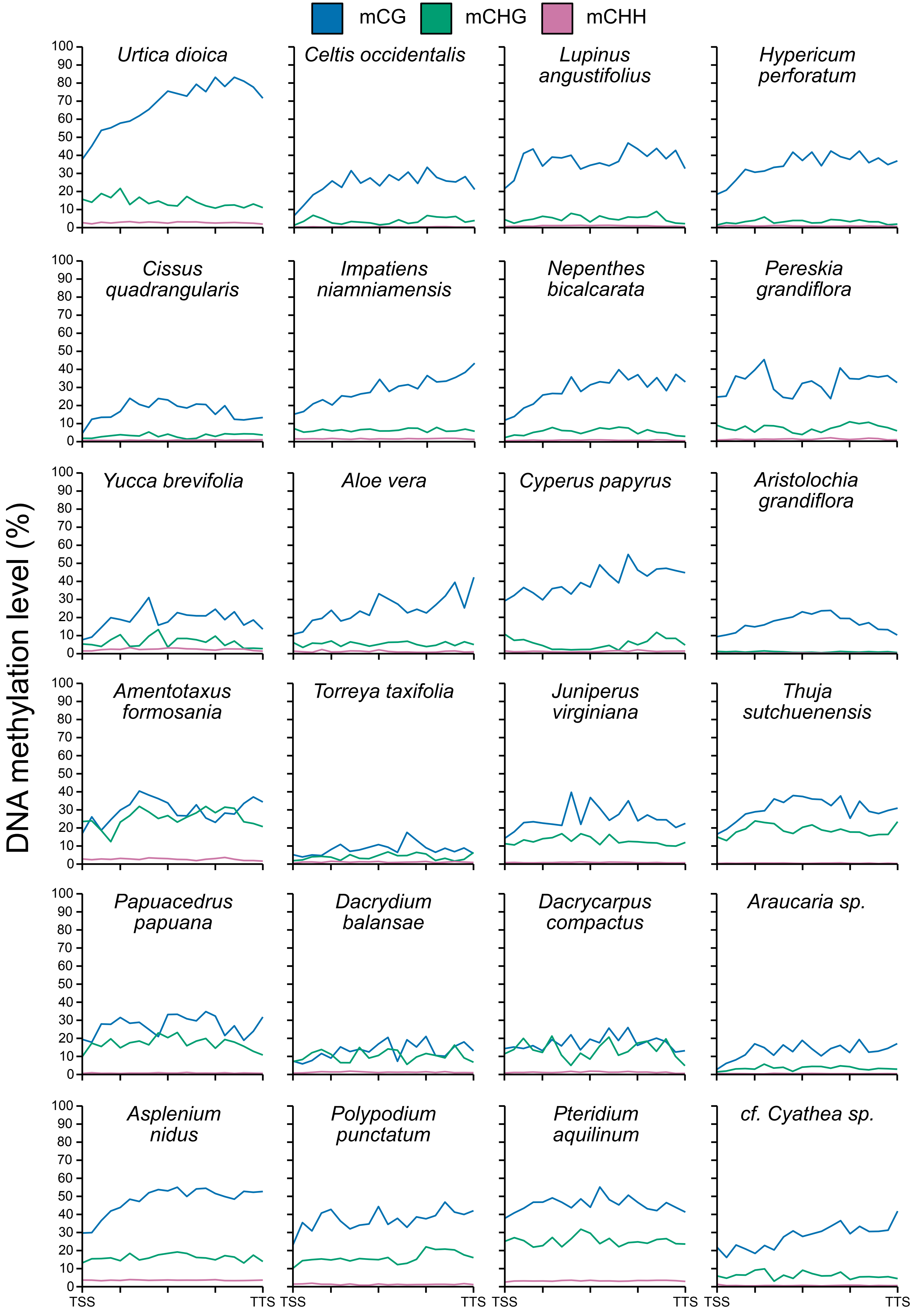

mCG
mCHG
mCHH
DNA methylation level (%)
Urtica dioica
Celtis occidentalis
Lupinus angustifolius
Hypericum perforatum
Cissus quadrangularis
Impatiens niamniamensis
Nepenthes bicalcarata
Pereskia grandiflora
Yucca brevifolia
Aloe vera
Cyperus papyrus
Aristolochia grandiflora
Amentotaxus formosania
Torreya taxifolia
Juniperus virginiana
Thuja sutchuenensis
Papuacedrus papuana
Dacrydium balansae
Dacrycarpus compactus
Araucaria sp.
Asplenium nidus
Polypodium punctatum
Pteridium aquilinum
cf. Cyathea sp.
0
10
20
30
40
50
60
70
80
90
100
TSS
TTS

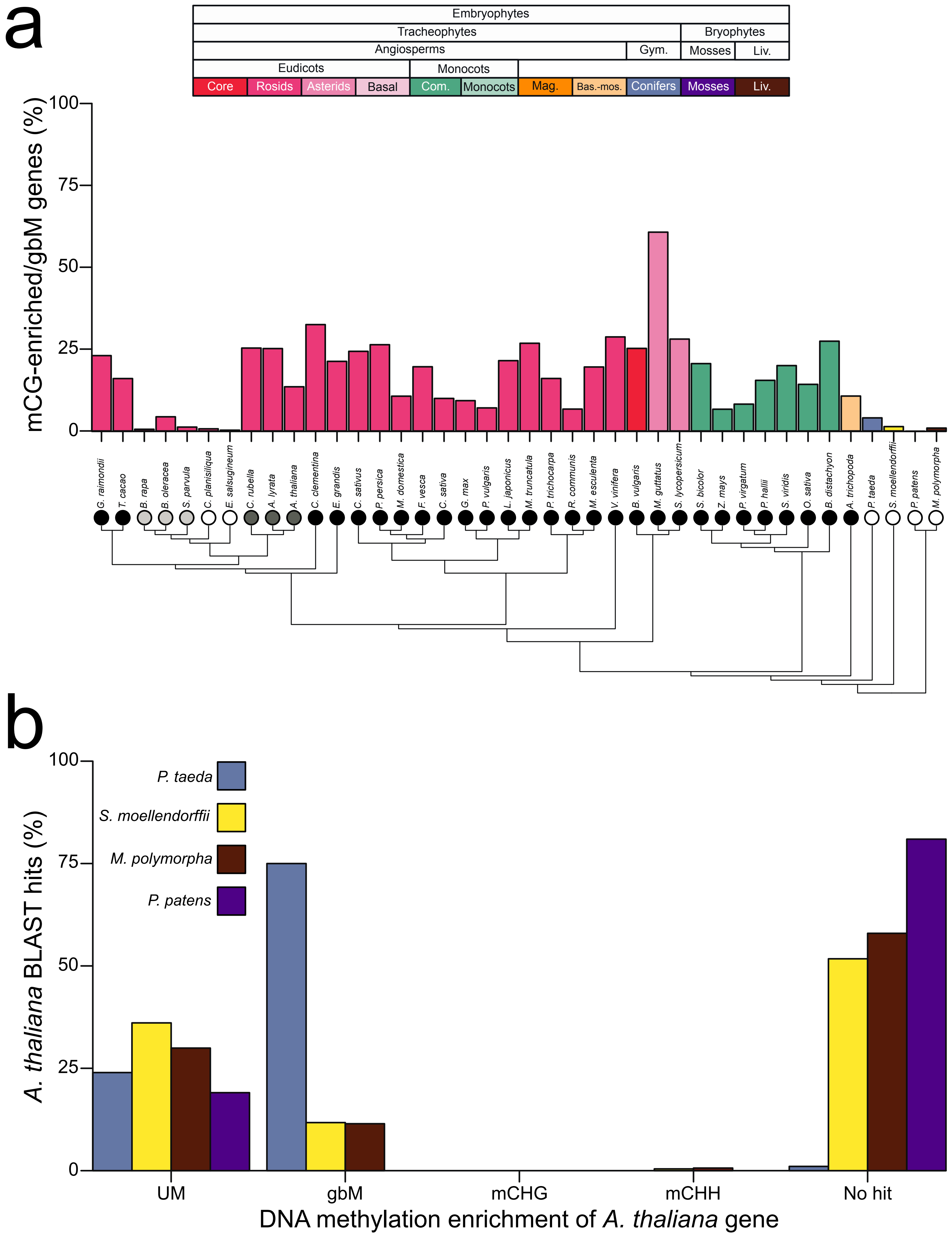
a
Embryophytes
Tracheophytes
Bryophytes
Angiosperms
Gym.
Mosses
Liv.
Eudicots
Monocots
Core
Rosids
Asterids
Basal
Com.
Monocots
Mag.
Bas.-mos.
Conifers
Mosses
Liv.
mCG-enriched/gbM genes (%)
100
75
50
25
0
G. raimondii
T. cacao
B. rapa
B. oleracea
S. parvula
C. planisiliqua
E. salsugineum
C. rubella
A. lyrata
A. thaliana
C. clementina
E. grandis
C. sativus
P. persica
M. domestica
F. vesca
C. sativa
G. max
P. vulgaris
L. japonicus
M. truncatula
P. trichocarpa
R. communis
M. esculenta
V. vinifera
B. vulgaris
M. guttatus
S. lycopersicum
S. bicolor
Z. mays
P. virgatum
P. hallii
S. viridis
O. sativa
B. distachyon
A. trichopoda
P. taeda
S. moellendorffii
P. patens
M. polymorpha
b
A. thaliana BLAST hits (%)
100
75
50
25
0
P. taeda
S. moellendorffii
M. polymorpha
P. patens
UM
gbM
mCHG
mCHH
No hit
DNA methylation enrichment of A. thaliana gene

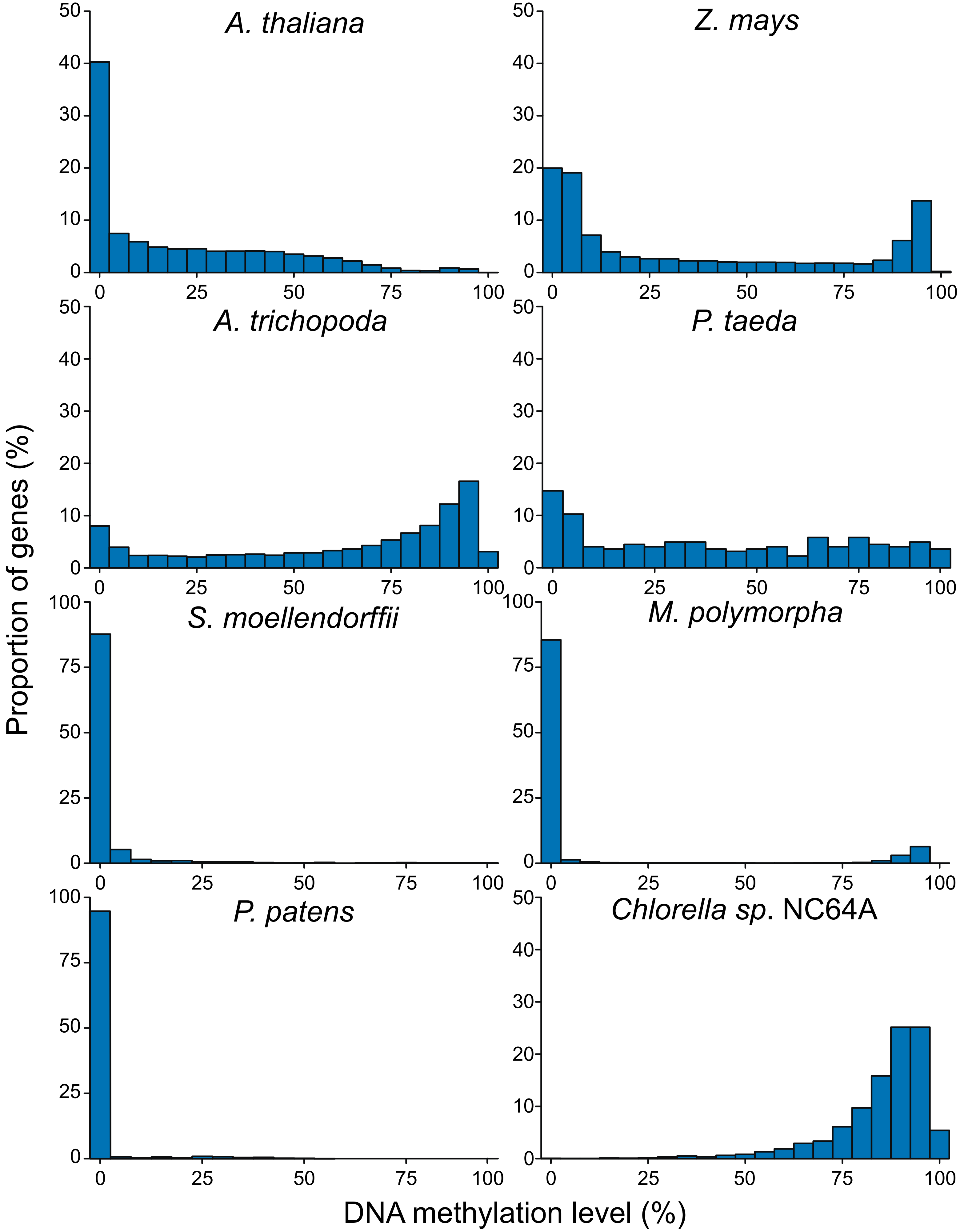

A. thaliana
Z. mays
A. trichopoda
P. taeda
S. moellendorffii
M. polymorpha
P. patens
Chlorella sp. NC64A
Proportion of genes (%)
DNA methylation level (%)
0
25
50
75
100
10
20
30
40

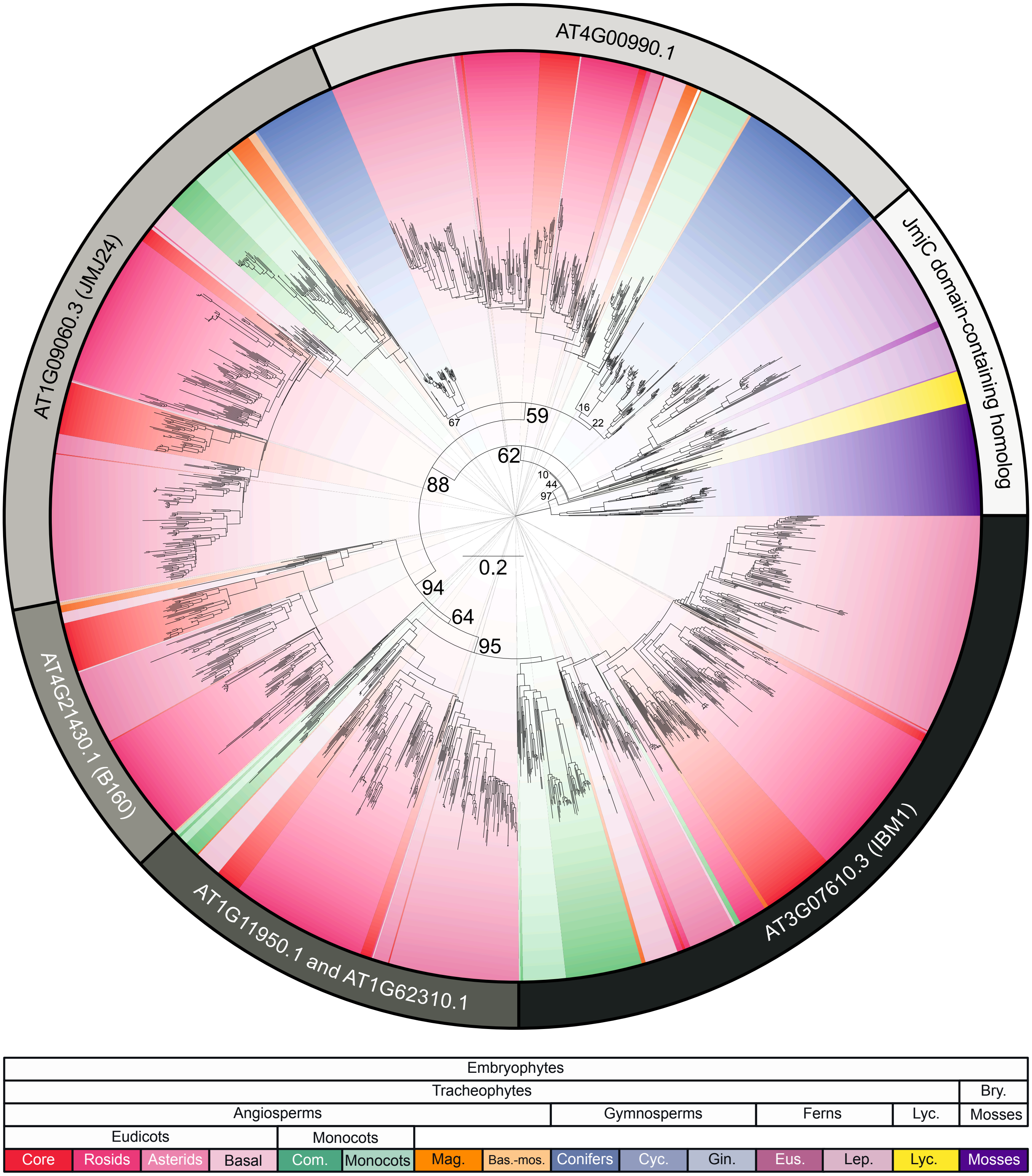
AT4G00990.1
JmjC domain-containing homolog
AT3G07610.3 (IBM1)
AT1G11950.1 and AT1G62310.1
AT4G21430.1 (B160)
AT1G09060.3 (JMJ24)
59
62
88
67
16
22
10
44
97
0.2
94
64
95
Embryophytes
Tracheophytes
Bry.
Angiosperms
Gymnosperms
Ferns
Lyc.
Mosses
Eudicots
Monocots
Core
Rosids
Asterids
Basal
Com.
Monocots
Mag.
Bas.-mos.
Conifers
Cyc.
Gin.
Eus.
Lep.
Lyc.
Mosses

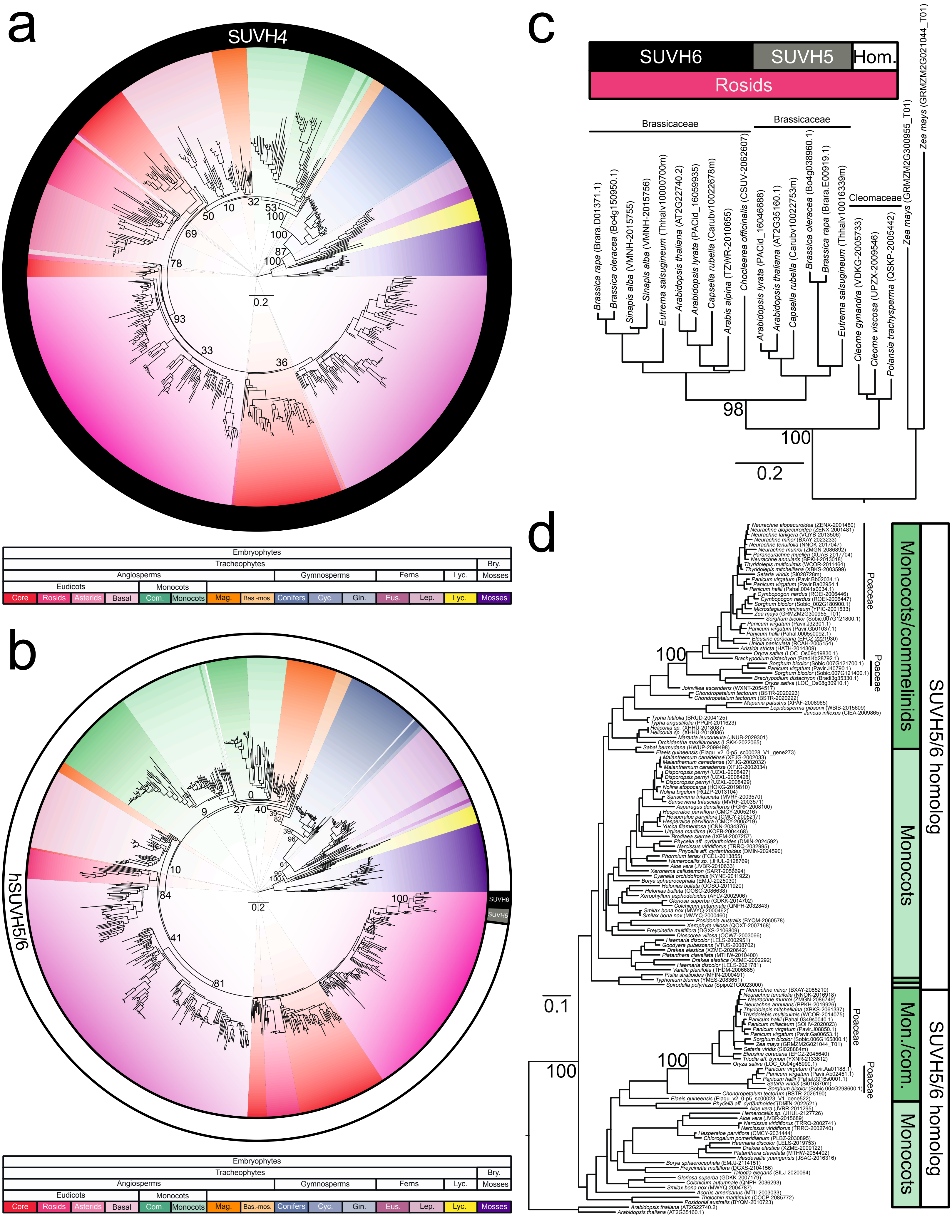
a
SUVH4
0.2
Embryophytes
Tracheophytes
Bry.
Angiosperms
Gymnosperms
Ferns
Lyc.
Mosses
Eudicots
Monocots
Core
Rosids
Asterids
Basal
Com.
Monocots
Mag.
Bas.-mos.
Conifers
Cyc.
Gin.
Eus.
Lep.
Lyc.
Mosses
b
hSUVH5/6
SUVH6
SUVH5
0.2
c
SUVH6
SUVH5
Hom.
Rosids
Brassicaceae
Brassicaceae
Cleomaceae
Brassica rapa (Brara.D01371.1)
Brassica oleracea (Bo4g150950.1)
Sinapis alba (VMNH-2015755)
Sinapis alba (VMNH-2015756)
Eutrema salsugineum (Thhalv10000700m)
Arabidopsis thaliana (AT2G22740.2)
Arabidopsis lyrata (PACid_16059935)
Capsella rubella (Carubv10022678m)
Arabis alpina (TZWR-2010655)
Choclearea officinalis (CSUV-2062607)
Arabidopsis lyrata (PACid_16046688)
Arabidopsis thaliana (AT2G35160.1)
Capsella rubella (Carubv10022753m)
Brassica oleracea (Bo4g038960.1)
Brassica rapa (Brara.E00919.1)
Eutrema salsugineum (Thhalv10016339m)
Cleome gynandra (VDKG-2005733)
Cleome viscosa (UPZX-2009546)
Polansia trachysperma (QSKP-2005442)
Zea mays (GRMZM2G300955_T01)
Zea mays (GRMZM2G021044_T01)
98
100
0.2
d
Monocots/commelinids
Monocots
SUVH5/6 homolog
Mon./com.
Monocots
SUVH5/6 homolog
Poaceae
Poaceae
Poaceae
Poaceae
100
100
100
0.1

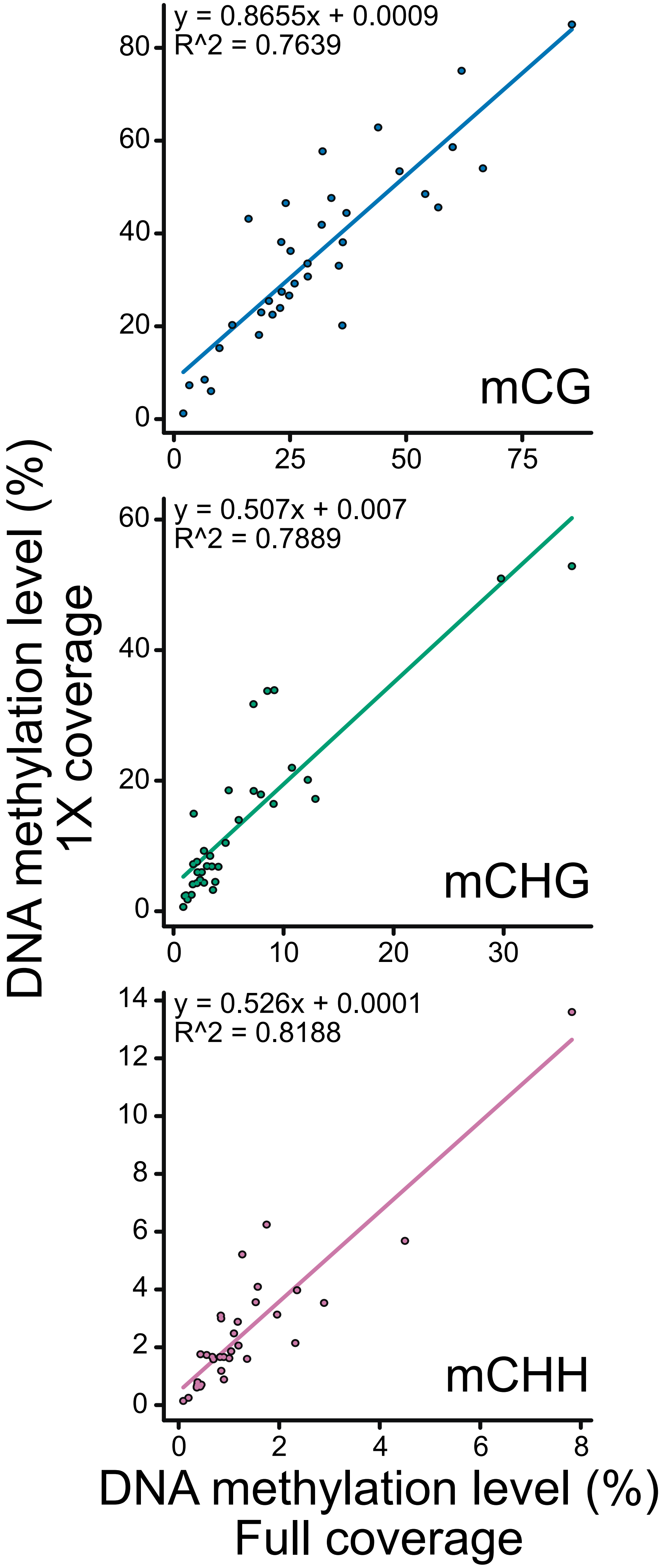

y = 0.8655x + 0.0009
R^2 = 0.7639
mCG
y = 0.507x + 0.007
R^2 = 0.7889
mCHG
y = 0.526x + 0.0001
R^2 = 0.8188
mCHH
DNA methylation level (%)
1X coverage
DNA methylation level (%)
Full coverage
